# Post-Insemination Infusion of Wharton’s Jelly Mesenchymal Stromal/Stem Cells-Derived Conditioned Medium: A Novel Approach for Improving Pregnancy Outcomes in Problem Mares

**DOI:** 10.3390/vetsci12050482

**Published:** 2025-05-16

**Authors:** Chiara Del Prete, Emilia Attolini, Barbara Merlo, Eleonora Iacono, Francesca Paola Nocera, Luisa De Martino, Consiglia Longobardi, Sara Damiano, Valentina Longobardi, Natascia Cocchia, Maria Pia Pasolini

**Affiliations:** 1 Department of Veterinary Medicine and Animal Production, University of Naples Federico II, 80137 Naples, Italy; chiara.delprete@unina.it (C.D.P.); francescapaola.nocera@unina.it (F.P.N.); ldemart@unina.it (L.D.M.); consiglia.longobardi@unina.it (C.L.); sara.damiano@unina.it (S.D.); valentina.longobardi@unina.it (V.L.); ncocchia@unina.it (N.C.); pasolini@unina.it (M.P.P.); 2 Department of Veterinary Medical Sciences, Alma Mater Studiorum-University of Bologna, 40064 Ozzano dell’Emilia, Italy; emilia.attolini2@unibo.it (E.A.); eleonora.iacono2@unibo.it (E.I.)

**Keywords:** equine, endometritis, mesenchymal stromal/stem cells, secretome, conditioned medium

## Abstract

Treating endometritis is one of the major challenges faced by veterinarians. The effect of post-insemination intrauterine infusion Wharton’s jelly mesenchymal stromal/stem cell-derived conditioned medium (WJ-MSC-CM) on the uterine response and pregnancy outcomes in problem mares was evaluated in this study. The cycles of 12 problem mares were treated seven/eight hours after insemination with intrauterine infusion of 20 mL of WJ-MSC-CM (TRT) or non-conditioned Ringer’s lactate solution (CTR). Before (PRE) and 12 h after (POST) treatment, an ultrasonographic examination and a low-volume flush (LVF) for efflux clarity evaluation and bacteriological and cytological analysis were made. Pregnancy diagnosis was performed at 14 days and confirmed at 60 days after ovulation. A decrease (*p* < 0.05) in polymorphonuclear neutrophils infiltration and a trend toward significance in LVF efflux clarity (*p* < 0.1) were observed in TRT cycles. The TRT (50%) had a higher pregnancy rate (*p* < 0.05) than CTR group (0%) at first cycle. Only a trend toward significance was observed comparing pregnancy frequencies between the two groups (CTR 12.5% vs. TRT 54.5%), the pregnancy rate of TRT cycles was comparable to that of normal mares (*p* > 0.05) and higher (*p* < 0.05) than that reported for problem mares in the literature. Post-insemination uterine infusion of WJ-MSC-CM in mares restores pregnancy rates to normal levels, by its anti-inflammatory effect.

## Simple Summary

Endometritis is the first cause of subfertility and the third most common disease affecting horses. The inconsistent effectiveness of traditional therapy and the rising incidence of antimicrobial resistance have heightened interest in alternative approaches. Therapies with Mesenchymal stem cells and their derivatives are increasingly recognized as promising options for treating uterine pathologies due to their immunomodulatory and antimicrobial properties. This study explored the possibility of treating problem mares with a post-insemination intrauterine infusion of Wharton’s jelly mesenchymal stromal/stem cell-derived conditioned medium, by evaluating its anti-inflammatory and antimicrobial properties, as well as pregnancy rates. Post-insemination therapy enhanced the anti-inflammatory response and the pregnancy rates, making them comparable to those of normal mares and higher than those reported for problem mares in the literature. In addition, although the elimination of bacteria was not observed 12 h after treatment, the successful pregnancy following this treatment suggests a potential antimicrobial role. Those findings lay the foundations for the establishment of new treatment protocols for uterine pathologies in mares and other species.

## 1. Introduction

Endometritis, an infection and/or inflammation of the endometrium, is the leading cause of subfertility and the third most common disease affecting horses [1,2]. It can have infectious and non-infectious etiologies, which often coexist in clinical practice [3]. Post-breeding inflammation occurs in all mares within 30 min after insemination [4,5]. This physiological response facilitates the clearance of seminal plasma, excess sperm, microorganisms, and debris from the uterine lumen in preparation for embryo implantation [6]. However, mares susceptible to endometritis exhibit a delayed onset and a prolonged uterine inflammatory response [7,8,9]. This leads to an excessive accumulation of polymorphonuclear neutrophils (PMNs) and intrauterine fluid persisting for up to 96 h or longer post-breeding, adversely affecting embryonic survival and pregnancy establishment [10,11]. While pregnancy rates range from 40% to 65% in healthy mares, they drop below 22% in cases of severe endometrial inflammation [12,13,14]. Given that the equine embryo migrates from the oviduct to the uterine lumen between 144 and 168 h after ovulation [15], inflammation must resolve within a limited timeframe. Moreover, a compromised innate immune response in affected mares hampers microbial clearance, increasing the risk of persistent infection and chronic inflammation [16]. Current treatment strategies aim to mitigate uterine inflammation and provide antimicrobial support, often in combination with mechanical clearance methods, such as uterine lavages and ecbolic administration [17,18,19]. However, conventional therapies demonstrate inconsistent efficacy, and the rising incidence of antimicrobial resistance has fueled interest in alternative approaches [19,20]. Regenerative therapies, including mesenchymal stromal/stem cells (MSCs) and their derivatives, are emerging as promising candidates due to their immunomodulatory and antimicrobial properties [21].

MSCs are self-renewing cells capable of differentiating into multiple mesodermal and ectodermal tissues. They secrete cytokines, growth factors, and extracellular matrix molecules that regulate hematopoiesis, angiogenesis, and immune and inflammatory responses. They are also involved in regenerating and remodeling injured tissues through their trophic activities, such as inhibiting apoptosis [22,23,24]. MSCs can be isolated from various tissues, including bone, cartilage, tendon, muscle, adipose tissue, and fetal membranes [25]. MSCs derived from birth-associated tissues, such as the placenta and the umbilical cord/Wharton’s jelly (WJ-MSCs), exhibit superior proliferative capacity, particularly under hypoxic conditions [26]. Although evidence remains limited, numerous studies have demonstrated the antimicrobial potential of MSCs both in vitro and in vivo [27,28,29]. This antimicrobial activity is attributed to the secretion of antimicrobial peptides (AMPs), which disrupt the cell membranes of bacteria, as well as fungi, yeasts, and viruses [30,31]. Additionally, MSCs exert indirect antimicrobial effects by modulating immune responses, enhancing phagocytosis, and regulating cytokine secretion [32]. Recently, MSCs-derived products, particularly conditioned medium, have gained interest as a cell-free therapy due to their safety, ease of storage, and transportability [33]. MSC-conditioned medium (MSC-CM) is a biological solution containing the secreted factors produced by MSCs during their growth in culture [34].

MSCs secretome includes microvesicles and exosomes, which mediate many of the therapeutic effects attributed to MSCs [35]. Importantly, MSC-CM provides similar anti-inflammatory and antimicrobial benefits while reducing risks such as immune rejection, tumorigenesis, and thrombosis [34,35,36].

This study aimed to evaluate the impact of post-insemination intrauterine infusion of Wharton’s jelly mesenchymal stromal/stem cell-derived conditioned medium (WJ-MSC-CM) on pregnancy outcomes in problem mares, by investigating antimicrobial and anti-/pro-inflammatory effects.

## 2. Materials and Methods

### 2.1. Animals

This study involved Standardbred mares from a single farm in the Campania Region (Italy), all reproductively managed by the same practitioners during the 2023 breeding season. Mares were selected based on the following criteria: (i) failure to conceive during the previous breeding season or after at least two insemination attempts with proven fertile semen in the same year; (ii) designation for artificial insemination with cooled semen from stallions of proven fertility; (iii) presence of endometrial inflammation or bacterial load during post-insemination evaluation; (iv) absence of other reproductive disorders as determined by transrectal palpation, ultrasound examination of the genital tract, and vaginal examination at the beginning of the study. A total of 13 mares were evaluated for inclusion in the study.

### 2.2. Study Design

Selected mares were homogenously assigned based on parity and age to either the control (CTR) or treatment (TRT) cycle. If they did not become pregnant, they were subsequently assigned to the alternate cycle for a maximum of two cycles per mare. If they became pregnant, they were not treated again.

The intrauterine infusions were performed 7–8 h after artificial insemination with cooled semen. Transrectal ultrasound evaluation of the reproductive tract for uterine edema and fluid accumulation and a uterine low-volume flush (LVF) was performed immediately before (PRE) and 12 h after (POST) intrauterine infusion. The LVF underwent bacteriological and cytological analyses, and interleukin-10 (IL-10) concentrations were also measured. Pregnancy was diagnosed by ultrasonography 14 days after ovulation and confirmed at 60 days.

### 2.3. Preparation of the Conditioned Medium

Umbilical cords (UCs) were collected immediately after foaling from mares hospitalized for attended delivery at the Perinatology and Reproduction Unit of the Equine Clinical Service, Department of Veterinary Medical Sciences (DIMEVET, University of Bologna, Italy). Only UCs from healthy mares with normal pregnancies, eutocic deliveries, and healthy foals were collected, as described by Lanci et al. (2023) [37].

Immediately after spontaneous rupture, the UC portion closest to the foal, characterized by an abundant amount of WJ, was collected. Samples were stored in Dulbecco’s phosphate-buffered saline (DPBS) containing 100 IU/mL penicillin and 100 mg/mL streptomycin, at 4 °C for at the latest maximum of 12 h, and transferred to the Animal Reproduction and Biotechnology Laboratory of the Equine Clinical Service (DIMEVET, University of Bologna, Bologna, Italy). MSCs were isolated from the Wharton’s Jelly and expanded in vitro following the protocols described by Iacono et al. (2012) [38], then frozen as described by Merlo et al. (2016) [39]. Cells were stored for at least 4 months in liquid nitrogen before thawing for conditioned medium preparation. WJ-MSCs were thawed, plated in T75 flask at a density of 1 × 10^6^ cells/flask, and cultured in Dulbecco’s Minimum Essential Medium (DMEM) supplemented with 10% fetal bovine serum (FBS) (Gibco, Thermo Fisher Scientific, Monza, Italy) until 80–90% confluence. Cells were then washed three times with DPBS and the culture medium was replaced with sterile Ringer’s Lactate solution (for IV administration, Galenica Senese, Monteroni d’Arbia, Italy). After 24 h of culture, the solution was recovered and centrifuged at 4000× *g* for 30 min at 25 °C, to remove cellular debris. The supernatant was collected as WJ-MSC-CM and frozen at −80 °C until use.

### 2.4. Insemination and Treatment

The estrous stage was monitored by transrectal ultrasound examination twice a week. In the presence of a pre-ovulatory follicle (35 × 35 mm) and endometrial edema (score 2/4 to 4/4 based on Rasmussen scoring system; score: 0: no edema, 1: mild edema, 2: moderate edema, 3: strong edema, and 4: excessive edema) [40,41], ovulation was induced with an intravenous injection of 2000 IU of human chorionic gonadotropin (Corulon^®^, Intervet Italia, Latina, Italy). Artificial inseminations with cooled semen from stallions of proven fertility, selected based on owner preference, were performed 24 h after ovulation induction using an artificial insemination catheter. After 7–8 h, mares received an intrauterine infusion of either 20 mL of Ringer’s Lactate (CTR group) or WJ-MSC-CM (TRT group) via a sterile insemination catheter.

Transrectal ultrasound evaluation and LVF were performed immediately before (PRE) and 12 h after (POST) intrauterine infusion. The presence of intrauterine fluid in the uterine body with maximum height greater than 2 cm, as well as uterine status was recorded [41]. Uterine edema was assessed using the Rasmussen scoring system ranging from 0 to 4 [40,41].

Before insemination, intrauterine infusion, and LVF, the perineal area and vulva were washed with warm water and scrubbed three times with povidone-iodine at 1% (Betadine^®^, Lombarda, Milan, Italy). LVF was performed using a sterile insemination cannula to introduce 120 mL of sterile Ringer’s Lactate into the uterus, which was then recovered in a sterile Falcon tube using 60 mL syringes. Macroscopic evaluation of the efflux clarity was performed immediately, scoring from 0 to 3 as described by LeBlanc (2011) [42], based on the amount of cells, mucus, or debris observed by rotating the tube while holding it up to the light. Samples were then transported to the laboratory at 4 °C within 8 h.

### 2.5. Analysis of LVF

In the laboratory, a portion of the LVF was aseptically extracted for the bacteriological examination, while the remainder was centrifuged. Bacteriological investigation was performed at the Bacteriology Laboratory of the Department of Veterinary Medicine and Animal Production (University of Naples “Federico II”), inoculating each aliquot in Brain Heart Infusion (BHI) broth and incubated aerobically at 37 °C for 24 h [43]. After the overnight incubation, turbid broths were streaked on different agar plates for Gram-positive and Gram-negative bacteria isolation (Columbia CNA agar with 5% sheep blood, Mac Conkey agar, Mannitol salt agar), which were incubated for additional 24 h at 37 °C. The bacterial identification was performed by using matrix-assisted laser desorption/ionization–time of flight mass spectrometry (MALDI-TOF MS) (Bruker Daltonics Inc., Bremen, Germany), following the manufacturer’s guidelines.

After cold centrifugation of the LVF (1000× *g* for 20 min), the supernatant was cryopreserved at −80 °C for evaluation within 2 months of the anti-inflammatory cytokine IL-10 concentration using a horse-specific ELISA kit (enzyme-linked immunosorbent assay) (Horse IL-10 ELISA kit, AssayGenie, Dublin, Ireland). The supernatant was first pre-treated to expose the antigen binding site and the resulting sample, appropriately diluted, was plated in a 96-well plate supplied by the kit and incubated at 37 °C. After 90 min, the Biotin-labeled antibody was incubated at 37 °C for 1 h. Following 3 washes with Wash buffer, the HRP-Streptavidin Conjugate was added to each well and incubated at 37 °C for 30 min. After 5 washes, TMB (5,5′-Tetramethylbenzidine) was added, and the plate was re-incubated at 37 °C for approximately 10 min, until a blue color developed. This color, upon addition of the Stop solution, turned yellow, and the plate was immediately read with a spectrophotometer (Thermo Fisher Scientific, Waltham, MA, USA) at 450 nm. IL-10 concentration, expressed in pg/mL, was calculated using a calibration curve.

For cytology, the pellet from the centrifuged LVF was collected with a swab and smeared onto two slides. The slides were stained using Diff-Quick staining (Bio-Optica, Milan, Italy) and evaluated with an optical microscope (Nikon Eclipse E600, Nikon, Tokyo, Japan). For each sample, 10 random fields at 1000× magnification (high power fields: HPF) were evaluated on each of the two slides, and the results were averaged [44]. Cells were categorized into endometrial epithelial cells, polymorphonuclear neutrophils (PMNs), and other inflammatory cells (eosinophils, lymphocytes, macrophages). Additionally, for each field, the presence of mucus, debris, and blood contamination (erythrocytes) was assessed, and the ratio of PMNs to total (epithelial) cells (PMN/EP) was calculated. Mares with 0–2 PMNs/HPF were classified as normal (0), as already reported by Riddle et al., [13,44]; those with 2 to 5 PMNs were classified as having moderate inflammation (1), while mares with more than 5 PMNs/HPF were classified as having severe inflammation (2).

### 2.6. Statistical Analysis

All results were collected in an Excel^®^ (Microsoft) file and then imported into IBM SPSS version 29 (IBM Corporation, Milan, Italy) for statistical analysis. The data were expressed as mean and standard deviation (SD). Uterine edema, efflux clarity, and inflammation scores, PMN/HPF, PMN/EP, and IL-10 concentrations were compared between cycles (CTR vs. TRT) before and after (PRE vs. POST) intrauterine infusion and within each cycle between PRE and POST using the Wilcoxon test. The frequencies of POST fluid accumulation and pregnancy in CTR and TRT cycles were compared with the Chi-square test or Fisher’s exact test for a small sample size. The binomial test was used to compare the expected with the observed frequency of positive pregnancies. Expected frequencies were calculated based on the literature [12,13,14], using 65% for normal mares and 22% for problem mares. Significance was set at *p* < 0.05, and tendencies were ascribed for *p* values between 0.05 and 0.1.

## 3. Results

**Figure 1:**
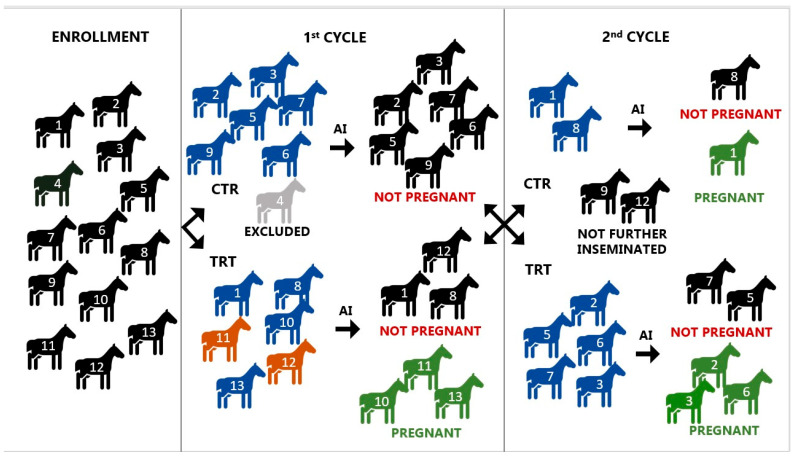
Schematic representation of the experiment, including mare assignment to either the control (CTR) or treatment (TRT) cycle and corresponding pregnancy outcomes. Each mare is identified by an individual ID number (1 to 13). Color legend: blue: positive bacteriology; orange: positive cytology gray: negative bacteriology and cytology; green: pregnant.

Figure 1 is a schematic representation of the experiment. A total of 12 mares, aged between four and twenty years (median 5), including six primiparous and six pluriparous, were selected; one mare (ID 4) was excluded due to the absence of endometrial inflammation or bacterial load during post-insemination evaluation.

Six mares were initially assigned to the CTR and the other six to the TRT. All mares initially assigned to CTR failed to become pregnant, and five out of these six were subsequently treated with WJ-MSC-CM (TRT). Of the six mares initially assigned to TRT, three became pregnant; of the remaining three, only two were later treated with unconditioned Ringer’s Lactate (CTR).

In seven out of twelve mares, both CTR and TRT cycles were carried out; of the remaining five, four underwent only TRT and one only CTR. Two mares (ID 9 and 12) that failed to become pregnant after CTR and TRT, respectively, were not assigned for a second CTR/TRT cycle due to management constraints and owner preferences.

As shown in Figure 1, a total of 19 cycles were included in the study; in 8 cycles, mares were infused with unconditioned Ringer’s Lactate (CTR), while in 11 cycles, mares were treated with WJ-MSC-CM (TRT).

### 3.1. Clinical Finding

The uterine edema scores significantly decreased between PRE and POST in both CTR (PRE 3.0 ± 1.2 vs. POST 2.0 ± 1.1, *p* < 0.05) and TRT cycles (PRE 3.0 ± 0.8 vs. POST 2.1 ± 0.9 *p* ≤ 0.01). Efflux clarity scores of LVF in CTR cycles were 2.7 ± 0.8 at PRE and 1.8 ± 1.3 at POST, whereas in TRT cycles, they were 2.0 ± 0.8 at PRE and 1.0 ± 1.1 at POST. No significant differences were found between CTR and TRT at either time point. However, while no differences were observed between PRE and POST in CTR cycles, TRT cycles showed a tendency toward significance (*p* = 0.06) for improved efflux clarity.

**Figure 2:**
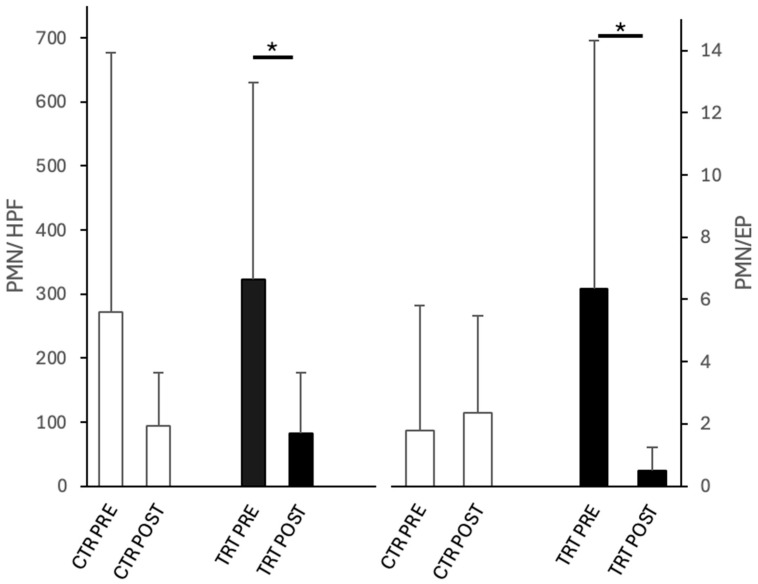
PMN/HPF and PMN/EP in the cytology of low volume flush of cycles before (PRE) and after (POST) intrauterine infusion of either unconditioned Ringer’s Lactate (CTR = 8) or Wharton’s jelly mesenchymal stromal/stem cell-derived conditioned Ringer’s Lactate (TRT = 11). Asterisks indicate significant differences at *p* < 0.05 (*) between groups within each time point or between time points within each group.

Endometrial inflammation scores evaluated by cytology were PRE 1.0 ± 1.1 vs. POST 1.3 ± 0.8 in CTR and PRE 1.5 ± 0.8 vs. POST 1 ± 0.9 in TRT, showing no differences between untreated and treated cycles in both PRE and POST time points. However, as shown in Figure 2, the number of PMN/HPF and the PMN/EP ratio significantly decreased (*p* < 0.05) between PRE and POST only in TRT cycles.

Uterine fluid was detected in POST evaluations in 12.5% (1/8) of CTR cycles and 18.2% (2/11) of TRT cycles, with no significant differences between groups.

### 3.2. Bacterial Isolation and Identification

Table 1 presents the bacteriology results of each mare in both control and treated cycles at PRE and POST and the pregnancy outcomes. For all isolated bacteria, MALDI-TOF MS identification gave a log(score) ≥ 2.0, confirming a highly reliable species identification. In control cycles, LVF culture PRE was positive in all cases, and remained positive in POST, with the same isolated bacteria; except in one case where two different bacterial species were isolated between PRE (*Deftia tsurunatensis*) and POST (*Escherichia coli*). In particular, *Escherichia coli* (*E. coli*) was the most detected bacterium (6/8; 75.0%), being isolated in both PRE and POST samplings from five out of eight mares (62.5%) and recovered only once alone (ID 8) in POST. Among the five mares, *E. coli* was isolated as a single bacterial species in two cases (ID 2, 6), whereas in three cases (ID 1, 3, 7), it was identified together with *Streptococcus equi* subsp. *zooepidemicus*, *Streptococcus equinus*, and *Enterococcus faecalis*, respectively. Among the eleven treated cycles, two were negative in both PRE and POST analysis, six tested positive for the same bacteria in both PRE and POST analyses, one case showed two different bacterial species isolated in PRE and POST, and two cycles were positive only in POST. Similarly, in treated cycles, *E. coli* was the most frequently identified bacterium (7/11; 63.6%). In contrast, the other recovered bacterial species exhibited a high degree of variability in isolation.

### 3.3. IL-10 Concentrations

**Figure 3:**
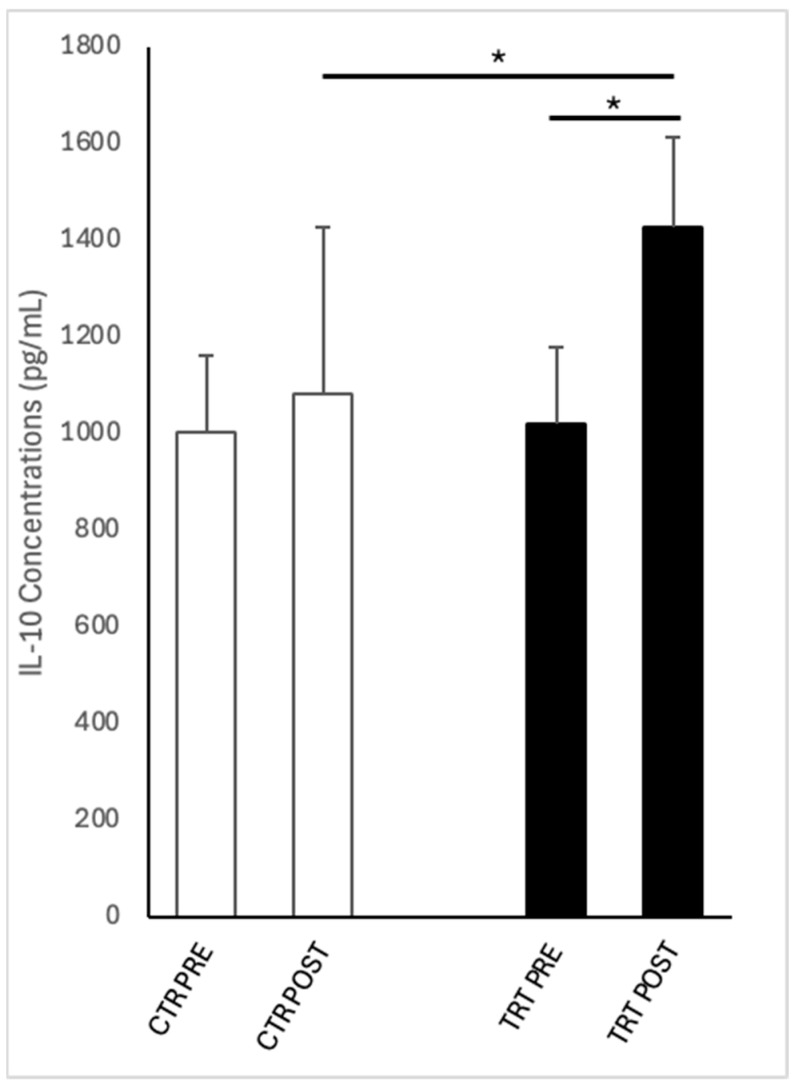
IL-10 concentrations in low volume flush of cycles before (PRE) and after (POST) intrauterine infusion of either unconditioned Ringer’s Lactate (CTR; n = 7) or Wharton’s jelly mesenchymal stromal/stem cell-derived conditioned Ringer’s Lactate (TRT; n = 9). Asterisks indicate significant differences at *p* ≤ 0.05 (*) between groups within each time point or between time points within each group.

Figure 3 shows IL-10 concentrations in the LVFs of control and treated cycles PRE and POST intrauterine infusion of either unconditioned or conditioned Ringer’s Lactate. Comparing PRE and POST, IL-10 concentrations in LVF increased (*p* < 0.05) following intrauterine infusion in TRT cycles but not in CTR ones. Furthermore, at the POST time point, IL-10 concentrations in LVF were significantly higher (*p* < 0.05) in the treated cycles compared to the CTR cycles.

### 3.4. Pregnancy Rates

Pregnancy outcomes for each cycle are presented in Figure 1 and Table 1. In the first cycle, the TRT group (3/6, 50.0%) had higher pregnancy rate (*p* < 0.05) than the CTR group (0/6, 0.0%), while no differences were found in pregnancy outcomes in the second cycle (CTR 1/2, 50.0% vs. TRT 3/5, 60.0%). A trend toward significance (*p* = 0.08) was observed when comparing overall pregnancy frequencies between TRT cycles (6/11, 54.5%) and CTR cycles (1/8, 12.5%). Similarly, considering only the presence of bacteria, a trend toward significance (*p* = 0.06) was observed when comparing pregnancy rates between TRT cycles (5/9, 55.6%) and CTR cycles (1/8, 12.5%).

When comparing the observed pregnancy frequencies with those reported in the literature as expected for normal mares, no differences were found for TRT cycles, whereas a significant difference was observed for CTR (*p* < 0.05). Moreover, the pregnancy frequency in CTR did not differ from those reported in the literature as expected percentages in problem mares, while the frequency in TRT was significantly higher than that of problem mares (*p* < 0.05).

## 4. Discussion

This study tested for the first time the therapeutic potential of intrauterine infusion of WJ-MSC-CM in the treatment of endometritis in mares.

MSCs and their derivatives have been recognized for their immunoregulatory properties, making them valuable in treating acute and chronic inflammations [34]. The properties of WJ-MSCs make them an alternative source of stem cells for regenerative medicine. Unlike embryonic stem cells, no ethical concerns are associated with their clinical application. Due to their embryonic nature, WJ-MCs exhibit higher expression of pluripotency markers such as NANOG, Oct 3/4, and SOX2 compared to adult MSCs [45,46]. Furthermore, WJ-MSCs demonstrate a higher proliferation rate, longer longevity, greater differentiation potential, immune privilege, and lower immunogenicity than adult MSCs [47]. It has been observed that MSCs from embryonic tissues, compared to those from bone marrow, enhance Treg cell activity and exhibit greater immunosuppressive effects [48,49]. The therapeutic effects of MSCs primarily occur through paracrine signaling of their secretome [50], which consists of soluble factors (growth factors, cytokines, chemokines, and enzymes) and extracellular vesicles (EVs) such as exosomes and microvesicles (MVs) containing lipids, proteins, RNA, and DNA subtypes [51]. Its benefits include minimizing the risk of survival issues or complications caused by improper differentiation of the cells within the host tissue, all while preserving their therapeutic potential [52,53].

MSCs-based therapies have been proposed for treating endometritis due to their ability to modulate the inflammatory response induced by the presence of sperm, debris, and bacteria during mating/insemination. In this study, the pregnancy rate following WJ-MSC-CM (54%) treatment was comparable to that of normal mares as reported in the literature, even if the difference between control and treated cycles only showed a trend toward significance. Indeed, the pregnancy rate in CTR remained at levels consistent with those reported for problem mares [12,13,14]. Positive results regarding pregnancy seem to be linked to the type of MSCs or derivatives used. Indeed, MSCs and their derivatives from perinatal tissues (embryo, placenta, umbilical cord) seem to have greater immunomodulatory properties than MSCs derived from adult tissues [54,55]. The beneficial effects of MSCs and their derivatives on fertility may be attributed to their role in facilitating paracrine communication between the conceptus and endometrium during early implantation and embryo development [56]. In cases of endometritis, MSCs release growth factors that enhance endometrial cell proliferation and stimulate angiogenesis, ultimately restoring fertility [57,58]. Prior studies have demonstrated that intrauterine administration of extracellular vesicles from amniotic MSCs over multiple cycles before-insemination in mares with chronic endometritis restored maternal–fetal communication and resulted in pregnancy [56]. Similarly, intrauterine therapy with MSCs of embryonic origin or their extracellular vesicles in mares susceptible to post-breeding induced endometritis has been associated with higher pregnancy rates [54].

The primary mechanism of action of WJ-MSC-CM observed in this study was an increase in IL-10 concentrations post-treatment, which was not detected in CTR. This is noteworthy because IL-10 is an essential anti-inflammatory cytokine for the immune response, capable of reducing the transcription of pro-inflammatory cytokines by monocytes and macrophages [59]. Our findings align with the literature, as MSCs from bone marrow, adipose tissue, and of amniotic origin have been demonstrated to induce IL-10 production both in vivo and in vitro [60,61,62]. MSCs’ administration has been demonstrated to reduce IL-6 concentration around 6 h post-insemination while increasing the levels of the anti-inflammatory cytokine IL-10 and IL-1RA in the uterus of susceptible mares at both 6 and 24 post-insemination [60]. As in our study, in a study by Lange-Consiglio et al. (2023) [61], the IL-10 concentration in the intrauterine fluid of the control group remained stable after insemination; on the contrary, the insemination with semen supplemented with extracellular vesicles from amniotic MSCs, IL-10 levels significantly increased 6 h post-insemination and remained unchanged up to 24 h. The increase in IL-10 and the decrease in IL-6 following MSC treatments confirm their mechanism of action: they can regulate the immune response via paracrine communication. Despite the different timings, the effect of MSC derivatives consistently appears to increase in IL-10 after insemination. Woodward et al. (2013) [11] demonstrated that 6 h after insemination, susceptible mares have a reduced expression of the anti-inflammatory cytokines IL-10 and IL-1RA (IL-1RN), which are necessary to resolve inflammation. The ability of intrauterine WJ-MSC-CM infusion post-insemination to increase IL-10 concentrations could help address this deficiency in problem mares.

Nearly all mares in this study exhibited moderate to severe endometrial inflammation on cytology at the time of the intrauterine infusion. The increase in IL-10 in treated cycles was associated with a reduction in PMN infiltration and PMN/EP ratio, along with a trend toward improved LVF efflux clarity. However, there was no significant reduction in inflammation score.

The lack of significant change in some inflammatory parameters may be attributed to the small sample size and the timing of treatment and sample collection. Previous studies have observed reductions in inflammatory markers when MSCs or their derivatives were administrated before insemination and evaluated 24 h post-insemination (i.e., 48 h after treatment). For instance, a decrease in intrauterine fluid accumulation was observed 24 h after insemination in mares treated with adipose-derived MSCs or extracellular vesicles derived from amniotic MSCs [54,62]. Ferris et al. (2014) [60] observed a reduction in PMNs and the cytokine IL-1 in the LVF of mares treated with MSCs 24 h before insemination. Positive outcomes on inflammation and fertility have also been observed following the addition of MSCs or conditioned medium directly into semen [61,62]. MSCs and their derivatives had no negative effects on semen and instead led to a reduction in PMNs in the endometrium, intrauterine fluid accumulation, and pro-inflammatory cytokines 24 h after insemination.

Previous reports have indicated that the intrauterine treatment of susceptible mares with MSCs or derivatives before or with insemination plays a positive role in immunomodulation, preventing excessive inflammatory responses and improving fertility [54,60,61,62]. It must be noted that MSCs and derivatives from different tissue types (adipose, bone marrow, placental) have been used in those studies, and a direct comparison of the results is not possible. However, our results represent the first step in supporting the hypothesis that mares can also be treated 6–8 h after insemination. To likely demonstrate a significant reduction in inflammation score, the LVF for cytology should be conducted at least 48 h after treatment.

The endometrial bacteria isolated in this study align with those commonly found in equine post-insemination endometritis: streptococci, coliforms, *Pseudomonas Aeruginosa*, and *Staphylococcus aureus* [18,63,64,65,66]. Among these, *Streptococcus equi* subspecies *zooepidemicus* and *E. coli* are the primary pathogens associated with endometritis [43,67,68,69,70] and, overall, *E. coli* was the most frequently identified bacterium in this study, consistently appearing across various samples and treatment conditions. Although bacteria were still isolated after treatment, almost all mares with positive bacteriology that received WJ-MSC-CM became pregnant, suggesting a potential antimicrobial effect.

Several studies have demonstrated the antimicrobial properties of MSC both in vitro and in vivo [27,31,33]. MSCs secrete antimicrobial peptides that disrupt bacterial membranes, inhibit nucleic acid synthesis, or interact with bacterial receptors [28]. Antimicrobial peptides such as cathelicidins, defensins, lipocaines, and hepcidins target bacteria, fungi, yeasts, and viruses [29]. MSCs also exhibit indirect antimicrobial mechanisms by coordinating pro- and anti-inflammatory elements of the immune system, increasing phagocytosis, and modulating cytokine secretion [32]. MSCs reduce the migration of pro-inflammatory cells and immunoregulatory factors, and express immunosuppressive molecules such as IL-17, which increases neutrophil phagocytosis, and indoleamine 2,3-dioxygenase (IDO) [29]. The latter works by degrading the essential amino acid tryptophan, necessary for pathogens’ proliferation, defeating bacterial replication and inducing a broad-spectrum antimicrobial activity [71]. Meisel et al. (2011) [31] evaluated the antimicrobial efficacy of MSCs in vitro, finding a broad-spectrum effect. Marx et al. (2020) [27] demonstrated that equine MSCs secretome is effective against bacteria in biofilms, including the antibiotic-resistant methicillin-resistant *Staphylococcus aureus*, via secretion of active proteases that destabilize biofilms by protein degradation, resulting in increased antibiotic effectiveness. The lack of negativization after WJ-MSC-CM infusion can be explained by the timing, as the WJ-MSC-CM may not have had sufficient time, 12 h, to reduce the bacterial load. In in vitro studies, a reduction in microbial load was observed after 16 and 24 h [27,31,72]. Cortés-Araya et al. (2018) [33] also demonstrated that in vitro, at least 16 h are required for the equine conditioned medium to reduce *E. coli* growth. The timing used in this study was dictated by field conditions, as it corresponded to the time of ovulation monitoring.

However, in three cases, we observed a different bacterial profile after WJ-MSC-CM intrauterine infusion (POST) compared to the profile identified before the infusion (PRE). Specifically, in two of these cases, the bacterial investigation conducted before the infusion (PRE) yielded negative results, indicating no detectable bacterial growth. However, the post-infusion (POST) samples from the same cases tested positive: one case (ID 8) showed growth of *E. coli* and *Streptococcus equi* subsp. *Zooepidemicus*, while the other (ID 9) showed growth of *E. coli* alone. In the third case (ID 7), the initial (PRE) bacterial investigation was positive for *Pseudomonas* spp., whereas the post-infusion (POST) sample tested positive for *E. coli*. It is not possible to determine whether the presence of these bacteria was an expected outcome of the infusion, as no change in microbial presence was observed in six other cases. This suggests that the detected bacteria may not be directly associated with the infusion procedure.

## 5. Conclusions

In conclusion, post-insemination uterine infusion of WJ-MSC-CM in mares restores pregnancy rates to normal levels. The primary mechanism of action of WJ-MSC-CM is its anti-inflammatory effect achieved through increased IL-10 concentrations, a cytokine essential for modulating the inflammatory response. Additionally, although no direct antimicrobial effect was demonstrated, the successful pregnancy WJ-MSC-CM-treated cycles suggests a potential antimicrobial role. Further studies are needed to confirm these findings and establish an optimal treatment protocol.

## Figures and Tables

**Table 1:** Bacteriological results of low volume flush from each control (CTR) and Wharton’s jelly mesenchymal stromal/stem cell-derived conditioned Ringer’s Lactate treated (TRT) cycles before (PRE) and after (POST) intrauterine infusion and pregnancy outcomes. Each mare is identified by an individual ID number (1 to 13).

Group	ID Mares	Cycle	PRE	POST	Pregnancy
CTR	2	1st	*Escherichia coli*	*Escherichia coli*	N
	3	1st	*Streptococcus equinus*; *Escherichia coli*	*Streptococcus equinus*; *Escherichia coli*	N
	5	1st	*Pseudomonas putida*	*Pseudomonas putida*	N
	6	1st	*Staphylococcus aureus*	*Staphylococcus aureus*	N
	7	1st	*Deftia tsurunatensis*	*Escherichia coli*	N
	9	1st	*Escherichia coli*	*Escherichia coli*	N
	1	2nd	*Streptococcus equi* subsp. *zooepidemicus*; *Escherichia coli*	*Streptococcus equi* subsp. *zooepidemicus*; *Escherichia coli*	Y
	8	2nd	*Enterococcus faecalis*; *Escherichia coli*	*Enterococcus faecalis*; *Escherichia coli*	N
TRT	1	1st	*Escherichia coli*; *Streptococcus equi* subsp. *zooepidemicus*	*Escherichia coli*; *Streptococcus equi subsp. zooepidemicus*	N
	8	1st	*-*	*Escherichia coli*; *Streptococcus equi* subsp. *zooepidemicus*	N
	10	1st	*Streptococcus equinus*	*Streptococcus equinus*	Y
	11	1st	*-*	*-*	Y
	12	1st	*-*	*-*	N
	13	1st	*Escherichia coli*; *Enterococcus faecalis*; *Staphylococcus aureus*	*Escherichia coli*; *Enterococcus faecalis*; *Staphylococcus aureus*	Y
	2	2nd	*Pseudomonas* spp.	*Escherichia coli*	Y
	3	2nd	*Escherichia coli*; *Staphylococcus schleiferi*	*Escherichia coli*; *Staphylococcus schleiferi*	Y
	5	2nd	*Streptococcus dysgalactiae*; *Klebsiella pneumoniae*	*Streptococcus dysgalactiae*	N
	6	2nd	*Staphylococcus aureus*; *Streptococcus dysgalactiae*; *Escherichia coli*	*Staphylococcus aureus*; *Streptococcus dysgalactiae*; *Escherichia coli*	Y
	7	2nd	*-*	*Escherichia coli*	N

CTR: control cycle; TRT: treated cycle; Y: yes; N: no.

## Data Availability

The raw data supporting the conclusions of this article will be made available by the authors on request.

## Abbreviations

The following abbreviations are used in this manuscript:

CM	Conditioned medium
CTR	Control group
DMEM	Dulbecco’s Minimum Essential Medium
DPBS	Dulbecco’s phosphate-buffered saline
*E. coli*	*Escherichia coli*
EP	Epithelial cells
HPF	High power field
IL-10	Interleukin-10
PMN	Polimorphonuclear neutrophils
POST	After treatment
PRE	Before treatment
TRT	Treated group
UC	Umbilical cord
WJ-MSC-CM	Wharton’s jelly mesenchymal stromal/stem cell-derived medium
